# Complete Genome Sequence of Paenibacillus polymyxa Strain Sb3-1, a Soilborne Bacterium with Antagonistic Activity toward Plant Pathogens

**DOI:** 10.1128/genomeA.00052-15

**Published:** 2015-03-12

**Authors:** Daria Rybakova, Ute Wetzlinger, Henry Müller, Gabriele Berg

**Affiliations:** Graz University of Technology, Institute of Environmental Biotechnology, Graz, Austria

## Abstract

The genome of Paenibacillus polymyxa Sb3-1, a strain that shows antagonistic activities against pathogenic fungi and bacteria, consists of one 5.6-Mb circular chromosome and two plasmids of 223 kb and 8 kb. The genome reveals several genes that potentially contribute to its antagonistic and plant growth promotion activity.

## GENOME ANNOUNCEMENT

Paenibacillus polymyxa, a Gram-positive endospore-forming bacterium, is considered to be a plant-growth-promoting rhizobacterium (PGPR) because several strains of this species have been identified as environmentally friendly alternatives to agrochemicals improving crop yield and quality (1). P. polymyxa strain Sb3-1 (syn. Paenibacillus kribbensis Sb3-1) was isolated from agricultural, organically managed soil in Egypt (2). The strain showed antifungal and antibacterial characteristics *in vitro* and was described as a potential biocontrol agent suppressing plant pathogens (3).

Genomic DNA of P. polymyxa Sb3-1 was isolated from an overnight culture using a DNeasy blood and tissue kit (Qiagen, Venlo, Netherlands) and was sequenced by PacBio RS technology using 8- to 12-kb inserts (GATC Biotech, Konstanz, Germany) yielding 102,991 reads with an average length of 5,597 bp; 91.6 percent of the total reads (94,101 reads) could be assembled using the *de novo* Hierarchical Genome Assembly Process (HGAP) implemented within the analysis pipeline SMRT Analysis 2.2 (Pacific Biosiences, CA, USA). The assembly resulted in three contigs representing the main chromosome and the two plasmids pSb31l and pSb31s with 82-fold overall coverage, whereas the obtained contigs from putative plasmids could be circularized with the AMOS package (4) and the chromosomal contig contained a gap of 2,050 bp. The gap was closed by designing specific primers matching with the ends of the genome and subsequent Sanger sequencing (LGC Genomics, Berlin, Germany). The whole-genome sequence of Paenibacillus polymyxa Sb3-1 consists of a main chromosome of 5,597,590 bp, and two plasmids, comprising 223,537 bp and 8,109 bp, respectively. The circular genome has a G+C content of 45.5%, and the plasmids pSb31s and pSb31l contained 45% and 42% of G+C nucleotides (RAST), respectively. Annotation was carried out automatically using the annotation software program BASYS (5) and the RAST server (6). Annotations derived from the RAST server were used for functional comparisons between different bacteria with a SEED viewer (7). The chromosome contained 5,168 coding sequences (CDS), 28 rRNA, and 109 tRNA loci. Plasmid pSb31s harbored seven putative genes, and one of them expressed a phage integrase family protein. The pSb31l plasmid contained 160 CDS as well as five rRNA genes.

We identified several genes putatively involved in antibiotic biosynthesis, such as genes encoding a bacitracin synthase 3, an iturin A synthetase, a bacillorin synthetase B, and a lantibiotic-synthetic gene cluster. In addition, genes responsible for nitrogen fixation were present in the P. polymyxa Sb3-1 genome as well as genes putatively involved in PGP, including those responsible for production of indole-3-acetic acid, 3-hydroxy-2-butanone (acetoin), 2,3-butanediol, and phytase. P. polymyxa Sb3-1 harbored at least seven nonribosomal peptide synthetase and five polyketide synthase gene clusters presented in several repetitions in the Sb3-1 genome.

This analysis provides the first insight into the mode of action of P. polymyxa Sb3-1 and will allow deeper understanding of the antagonistic properties and its interaction with the host.

### Nucleotide sequence accession numbers.

The genomic DNA of P. polymyxa Sb3-1 and the DNA of plasmids pSb31s and pSb31l have been deposited in DDBJ/ENA/GenBank under the accession numbers CP010268, CP010269, and CP010270, respectively.
